# Using the Medical Research Council Framework for the Development and Evaluation of Complex Interventions in a Theory-Based Infant Feeding Intervention to Prevent Childhood Obesity: The Baby Milk Intervention and Trial

**DOI:** 10.1155/2014/646504

**Published:** 2014-07-21

**Authors:** Rajalakshmi Lakshman, Simon Griffin, Wendy Hardeman, Annie Schiff, Ann Louise Kinmonth, Ken K. Ong

**Affiliations:** ^1^MRC Epidemiology Unit, University of Cambridge, Addenbrooke's Hospital, P.O. Box 285, Cambridge CB2 0QQ, UK; ^2^UKCRC Centre for Diet and Activity Research, University of Cambridge, Cambridge CB2 0QQ, UK; ^3^The Primary Care Unit, Department of Public Health and Primary Care, University of Cambridge, Cambridge CB2 0SR, UK; ^4^Department of Paediatrics, University of Cambridge, Cambridge CB2 0QQ, UK

## Abstract

*Introduction*. We describe our experience of using the Medical Research Council framework on complex interventions to guide the development and evaluation of an intervention to prevent obesity by modifying infant feeding behaviours.* Methods*. We reviewed the epidemiological evidence on early life risk factors for obesity and interventions to prevent obesity in this age group. The review suggested prevention of excess weight gain in bottle-fed babies and appropriate weaning as intervention targets; hence we undertook systematic reviews to further our understanding of these behaviours. We chose theory and behaviour change techniques that demonstrated evidence of effectiveness in altering dietary behaviours. We subsequently developed intervention materials and evaluation tools and conducted qualitative studies with mothers (intervention recipients) and healthcare professionals (intervention deliverers) to refine them. We developed a questionnaire to assess maternal attitudes and feeding practices to understand the mechanism of any intervention effects.* Conclusions*. In addition to informing development of our specific intervention and evaluation materials, use of the Medical Research Council framework has helped to build a generalisable evidence base for early life nutritional interventions. However, the process is resource intensive and prolonged, and this should be taken into account by public health research funders. This trial is registered with ISRTCN: 20814693 Baby Milk Trial.

## 1. Introduction

While the aetiology of obesity has been simplified to an imbalance between energy intake and energy expenditure over a prolonged period, the psychological, social, physiological, environmental, and other factors causing this imbalance are complex [1]. A complex problem does not always necessitate a complex intervention, but complex interventions, targeting multiple causal factors and the interactions between them, may be necessary [2]. Complex interventions are often not systematically developed, specified, or reported [3]. Following a systematic process in the development and evaluation of a complex intervention may help in understanding the processes underlying any observed intervention effects and for whom and in which settings interventions work, to inform and improve the development, evaluation, and implementation of future interventions.

**Figure 1:**
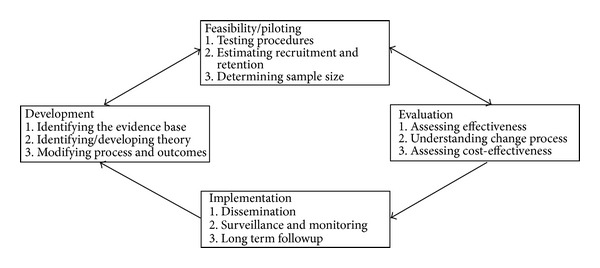
Key elements of the development, evaluation, and implementation process of complex interventions. Source: [2].

To address some of the complexities in defining, developing, and evaluating complex interventions, a number of frameworks have been proposed. These include Intervention Mapping [4, 5], RE-AIM (reach, efficacy, adoption, implementation and maintenance) [6, 7], Precede-Proceed [8], and Logic Models [9, 10]. More recently, the Medical Research Council (MRC) framework for developing and evaluating complex interventions originally published in 2000 [11] and updated in 2008 [2] has been recommended (Figure 1). The 2000 MRC framework suggested a model based on the phases conventionally used in the evaluation of new drugs—from initial preclinical research through to postmarketing surveillance [11]. The updated 2008 MRC framework provides a more flexible, less linear model of the process with greater attention to early phase piloting and development work [2].

The aim of this paper is to describe our experience of using the 2008 MRC framework to develop and evaluate a theory-based, behavioural infant feeding intervention aimed at preventing childhood obesity, including benefits and challenges of using this framework.

## 2. Methods

The activities we undertook and the stages of the 2008 MRC framework they map onto are shown in Table 1 and presented in more detail below.

### 2.1. Developing a Complex Intervention

#### 2.1.1. Identifying the Evidence Base

*Review of the Epidemiological Evidence for Early Life Risk Factors Contributing to Childhood Obesity*. The review highlighted the importance of excess energy intake resulting in excess weight gain during infancy [12–14], formula-feeding, and poor weaning practices in the development of obesity [15]. Randomised trials in small-for-gestational age and preterm infants showed that greater dietary energy content increased risk of obesity and metabolic disease in later life [16, 17]. In 2004, the World Health Organization and other international bodies reduced the recommended average energy requirements (AER) for infants by around 15 to 20% [18] and these energy requirements form the basis of the Baby Milk intervention. Babies who are fed formula-milk are more likely to show rapid weight gain during infancy than breastfed babies [19], possibly as a result of higher energy intake. In addition their mothers have a number of demographic characteristics that are associated with obesity risk (lower age, education, and socioeconomic status) and hence the intervention targets formula-fed babies (54% at 1 week age and 77% at 6 weeks age in UK [20]) through their mothers.

*Systematic Review of Parent's Experiences of Bottle-Feeding. *Having identified formula-fed babies as a high-risk group and excess energy intake amongst this group as a potential target for intervention, we sought to increase our understanding of the behaviours associated with excess energy intake by conducting a systematic review of the quantitative and qualitative literature around parents' experiences of bottle-feeding [21]. The review suggested that mothers who bottle-fed experienced negative emotions such as guilt, anger, worry, uncertainty, and a sense of failure. This emphasised the need for our intervention to be delivered with empathy and in a collaborative participant-centred style. Mothers reported receiving little information on bottle-feeding and did not feel empowered to make decisions. Mistakes in feed preparation and frequent formula-milk changes were common.

*Systematic Review of Determinants of Early Weaning. *We undertook a systematic review of the determinants of early weaning and inappropriate introduction of cow's milk to increase our understanding of why parents do not follow infant feeding recommendations [22]. Strong evidence was found for six maternal determinants of early weaning: young age, low education and socioeconomic status, absence/short duration of breastfeeding, smoking, and lack of information or advice from healthcare providers. The results of this review mirror the much larger body of evidence on determinants of breastfeeding [23–25]. Of these determinants, improving the advice and support given by healthcare providers appeared to be the area most amenable to intervention in the short term.

*Systematic Review of Interventions to Prevent Obesity in Young Children. *A 2011 Cochrane review on interventions to prevent obesity in children and adolescents identified 55 studies [26]. Only eight of these were targeting children aged 0–5 years and these studies showed the largest intervention effects, but none were specific to infancy. A search for “childhood obesity prevention” trials listed on registers of active and archived controlled trials (http://www.clinicaltrials.gov and http://www.controlled-trials.com) was conducted and nine trials in this age group were identified [27–35]. However none of them targeted energy intake from formula-milk, the focus of the baby milk intervention.

#### 2.1.2. Identifying/Developing Appropriate Theory

A number of psychological factors (e.g., beliefs and emotions) and environmental factors are involved in learning new behaviours and changing existing behaviours. Theories or models provide an overarching framework for the psychological and environmental factors that explain behaviours to be targeted by an intervention. As there were no behavioural interventions specifically targeting formula-milk feeding, this stage included a review of the literature on psychological theories and behaviour change techniques that had shown some success in improving dietary behaviours. 

*Social Cognitive Theory (SCT). *We identified Bandura's social cognitive theory [36] as a useful theory to inform mediators along the hypothesised causal pathways of intervention effects. The theory has been shown to predict other dietary behaviours, including fruit and vegetable intake in children [37], and has been used to develop interventions to improve breastfeeding practices [38] and dietary behaviours in adults [39, 40].

The key constructs of SCT are as follows.

*Perceived Self-Efficacy*. This refers to a person's belief or confidence in their ability to successfully perform a specific task or behaviour. High perceived self-efficacy is related to a feeling of being “*in control*” and a belief that “*I will be able to continue to perform the behaviour even in the face of difficult obstacles or stressful situations.*” 

*Outcome Expectancies*. These are person's thoughts or beliefs about the results or consequences of certain behaviour. Outcome expectancies can be either positive or negative and may be related to physical, social, and self-evaluative outcomes, that is, outcomes related to physical health, feedback from others, and feelings about oneself.

*Sociostructural Factors*. Environmental factors are referred to as sociostructural factors. These include all the factors outside of the person that might affect their ability to perform the target behaviour but are not necessarily beyond the person's control. An example of a sociostructural facilitator might be a local support group. A sociostructural barrier might be a grandparent or child minder unwilling to follow recommendations. Hence, the identification of barriers and facilitators for performance of the behaviour are techniques used in the intervention (Table 2).

*Implementation Intentions (IIs). *While SCT is promising in terms of strengthening motivation, it has been shown that good intentions do not always translate to behaviour change; hence we added implementation intentions (IIs). IIs have been shown to bridge the gap between motivation and action [41]. They commit an individual to a specific course of action when certain environmental conditions (barriers or facilitators) are met. The environment therefore acts as a cue to action and helps the individual to achieve their goal. IIs are “if…then…plans” specifying when, where, and how the person will act on their intentions and perform the behaviour and link the behaviour to specific cues [42].

*Behaviour Change Techniques (BCTs). *While theories provide a framework to understand how behaviours targeted in the intervention change, behaviour change techniques (BCTs) constitute the active content of interventions. We selected behaviour change techniques (BCTs) informed by the theoretical basis of our intervention (SCT and IIs) with evidence of effectiveness in changing dietary behaviours [43]. We used Abraham and Michie's taxonomy [44] to define the BCTs and operationalize them as intervention strategies in the intervention protocols (Table 2). The intervention aims to encourage parents to reduce the amount of formula-milk feeds, recognise infant satiety cues, not to respond to nonhunger related fussiness by feeding, wean babies onto a healthy diet, and recognise if their babies are gaining excess weight.  Table 3 summarises the contacts during which core intervention contents are used.

*Qualitative Studies. *Following initial development of the intervention, qualitative studies were conducted to assess the acceptability and feasibility of intervention delivery and appropriate changes to the intervention materials were made. Psychologists, dieticians, and doctors were interviewed and in addition, interviews and focus groups were conducted with relevant stakeholders-mothers (recipients of the intervention) and healthcare providers (health visitors and midwives who would deliver the intervention). An iterative process was used to refine the intervention [45]. One example of how this work informed intervention development is that “healthy growth” rather than “obesity prevention” was emphasised in the resources and communication messages. Furthermore, mention of breastfeeding being best was removed as mothers said this was not appropriate for a formula-feeding intervention. The studies also highlighted the need for repeated contacts delivered in an empathic, nonjudgemental, client-centred communication style and supported by written materials.

#### 2.1.3. Modelling Process and Outcomes

**Figure 2:**
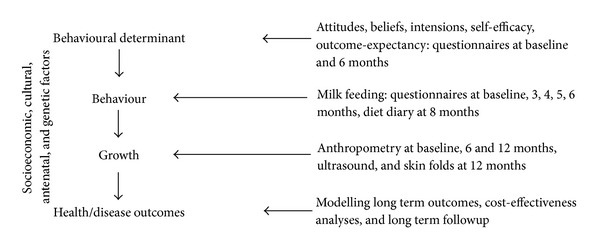
Hypothesised causal pathways and measures for evaluation in the Baby Milk trial.

A causal modelling approach [46] was used to link behavioural determinants causally through behaviour to physiological variables and health outcomes. Process and outcomes measures were mapped onto the causal pathway (Figure 2).

While validated measures existed to assess most variables along the causal pathway, we had to develop and validate a questionnaire to assess changes in the key behavioural determinants targeted by the intervention (maternal attitudes, SCT constructs targeted by the intervention, and milk feeding behaviour) [47]. The questionnaire showed good reliability (% agreement above 70% for 51/57 items, Kappas 0.37–1) and reasonable validity (% agreement above 66% for 39/57 items) and internal consistency (Cronbach's alphas 0.51, 0.79, and 0.90 for self-efficacy, outcome expectancies, and intention, resp.). Development of the questionnaire also influenced our thinking about intervention content.

### 2.2. Assessing Feasibility and Piloting Methods

#### 2.2.1. Testing Procedures

Once developed, all the intervention materials were piloted by the intervention facilitators (trained to deliver the intervention) to ensure that the intervention was acceptable and feasible to deliver. An extensive training manual and a two-day training programme in the evidence base underlying the intervention, theories, behaviour change techniques, intervention strategies, and communication skills, including demonstration and practice with individual feedback, were piloted and adapted. For example, we initially developed long versions of intervention protocols, however, during piloting, it became clear that it was difficult for intervention facilitators to use these and we developed shorter versions with key aspects of delivery. We piloted and refined checklists for each contact with intervention and control participants to be used by the intervention facilitators to assess and promote fidelity of intervention delivery (i.e., consistent delivery across facilitators and time). For the control group participants (attention control), in order to avoid contamination, we designed protocols with questions for each contact which were organised around broad themes (Table 3).

A 1-year pilot study (March 2011–March 2012) among 78 participants provided the opportunity to engage with local providers of postnatal and primary care services in order to optimise methods for recruitment, to assess the acceptability and feasibility of the trial measures, estimate expected retention rates, and plan the resources needed for an explanatory RCT. We did not analyse the results of the pilot feasibility study separately as it continued into the full trial.

#### 2.2.2. Estimating Recruitment and Retention

Our initial strategy to identify participants was to approach parents who were formula-feeding their baby before eight weeks of age via postnatal (midwives) and community health professionals (health visitors). To this end we spoke with midwives, infant feeding coordinators, and health visitors at their team meetings. Although this did not prove a very effective route for recruitment, partly due to the time pressures and conflicting priorities that these health professionals are faced with, it did help to raise awareness of the study and allowed us to collect information on ways to optimise recruitment and retention. For example, we extended the age of recruitment to 14 weeks, expanded our recruitment area, and offered easily accessible local clinics and/or home visit appointments.

After investigating a number of other strategies to identify participants including posters in health centres and children's centres, pharmacies, charity groups, and a local press release, we successfully applied for ethics approval for named members of our own research team to approach bottle-feeding mothers on postnatal wards directly. Since all babies are seen by their GPs for a six-week check, we also approached GPs for help with recruitment. In addition, participants were identified through the central health electronic database where a record of whether mothers were breastfeeding or bottle-feeding was made by their health visitor and a recruitment leaflet mailed to them. This multilevel approach from different professionals at different times during the first three months of infant age seemed to work well and indicated feasibility of recruitment. We did not offer any financial incentives but this could be a more effective way of recruiting, especially the hard-to-reach group and could be considered in future studies. Ongoing data on retention was monitored and barriers to retention identified.

#### 2.2.3. Determining Sample Size

The primary outcome was change in weight standard deviation score (SDS) from birth to age 12 months in intervention versus control groups. As there were no previous trials in this age group, we used data from observational studies of infant energy intake and growth to calculate the sample size and estimated that the target 15% lower energy intake would lead to a 0.30 SDS difference in weight [48]. Allowing for a 15% drop-out rate we needed to recruit 700 babies.

### 2.3. Evaluating a Complex Intervention

#### 2.3.1. Assessing Effectiveness

We decided that the most appropriate design to evaluate the effectiveness of this behavioural intervention would be a single (assessor) blind, individually randomised controlled trial. In order to assess true “intervention” effects, we decided the control group should get the same attention as the intervention group (attention control) and offered standard advice. Due to the paucity of research in this area, in addition to assessing whether the intervention was effective, we conducted a process evaluation to improve our understanding of the determinants of infant feeding behaviours, potential causal mechanisms underlying any intervention effects observed, implementation of this complex intervention, and contextual factors [49].

#### 2.3.2. Understanding Change Processes

The process evaluation included intervention fidelity assessment (implementation), a qualitative study and possibility for mediation analysis to illuminate contextual factors.

*Implementation. Fidelity Assessment. *In the Baby Milk trial, standard protocols for intervention and control group delivery were used for each contact. All planned facilitator-parent contacts (in both arms of the trial) were audiorecorded in order to assess fidelity of intervention delivery. Fidelity was promoted and contamination across the two groups minimised by assessing a random sample of audiotaped contacts using standardised fidelity checklists, followed by feedback, ongoing support over the whole period of intervention delivery, booster training sessions, and peer appraisal.

*Contextual Factors. *A Qualitative interview study among intervention (*n* = 10) and control (*n* = 10) group mothers and intervention facilitators (*n* = 4) explored how feeding decisions were made, to explain intervention effects, identify key ingredients that could be included in future interventions and identify contextual factors associated with variations in outcomes across participants.

Structural equation modelling can be used to test the complex relationships between mediators and outcomes, and paths through which they may exert their influence, bearing in mind the possibility of reverse causality where behaviour affects beliefs as well as vice versa [50]. For example, on the basis of SCT we hypothesize that beliefs about the health benefits for the child of following feeding recommendations (outcome expectancies) will partially mediate (explain) the relationship between confidence (self-efficacy) about following feeding recommendation and the formation of a goal.

#### 2.3.3. Cost-Effectiveness Analyses

In order for the results to be useful to decision makers, we developed instruments to collect cost-related data, time spent in delivering the intervention and health service utilisation. The analysis plan included a cost-consequences analysis to show the cost of delivering the intervention and outcomes (proportion of infants whose weight crosses more than one centile band upwards on the growth charts (0.67 SDS) and infants of normal weight at 12 months, and probability of being normal weight as an adult using data from a meta-analysis [51]), for intervention and control groups.

### 2.4. Implementation and Beyond

#### 2.4.1. Dissemination

A criticism of many trials is that their published reports do not describe the interventions in enough detail to enable them to be reproduced [52]. At the end of the trial we will make our training materials, intervention protocols, and fidelity checklists available on our website for other researchers to adapt and use.

Parents in the study and healthcare professionals who identify potential participants receive regular newsletters with key findings as they emerge. Results will be published in open access journals and reported to funders and policy makers.

#### 2.4.2. Surveillance, Monitoring, and Long-Term Followup

Ethical permissions and consent were taken to allow future recontacting of participants and/or accessing routinely collected data.

## 3. Discussion

### 3.1. Main Findings

Use of the 2008 MRC framework has helped develop a theory- and evidence-based intervention, to specify a proposed causal pathway to change infant feeding behaviour and growth outcomes, to pilot the intervention and study procedures in order to address the main uncertainties, and to design an explanatory RCT evaluation. Careful attention to the design of the RCT means the results not only will generate evidence about the effectiveness of a replicable intervention but also will allow us to begin to elucidate the processes by which change is achieved (or why it is not). Evaluations that take account of complexity of interventions could explain outcomes better even in the absence of intervention effectiveness [53, 54].

### 3.2. What Is Already Known on This Topic?

A number of frameworks have been proposed to aid researchers developing and evaluating complex interventions. The 2008 MRC framework suggests a comprehensive and iterative process for intervention development and evaluation.

### 3.3. What This Study Adds?

This paper explicitly maps the various activities and developmental and piloting work to the stages of the 2008 MRC framework [2] demonstrating how the framework can be operationalised. The greater emphasis on piloting and feasibility testing in the revised MRC framework is a strength as, in our experience, intervention content and materials, evaluation tools, and recruitment strategies were significantly improved through this process.

### 3.4. Limitations of This Study/Framework

Using the MRC framework posed a number of challenges, the biggest being the time and resources needed. Significant resources go into the development of pharmacological and other biomedical interventions, but the development of public health interventions which do not involve the generation of intellectual property does not receive such funding. This should be something funding bodies need to consider if public health interventions are to follow the same rigorous development and evaluation process that is used in drug development. With the current model of funding, it is very difficult for researchers in most countries to use the framework due to the timescales and resources required. Consequently the evidence base may be skewed towards “high income” countries where resources for development work may be more readily available. It could be argued that the process could be shortened and some of the stages omitted, especially if the evidence-base for what is likely to work is strong. Future evidence synthesis could focus on whether studies using the MRC framework are more effective than those not using it or using other frameworks.

## 4. Conclusions

Careful attention to intervention development is likely to result in interventions which advance the evidence base and may be a more efficient use of limited public health research resources.

## Figures and Tables

**Table 1:** Studies undertaken mapped to the phases of the MRC framework [2].

Definition	Studies undertaken
(1) *Developing a complex intervention *	

(1.1) *Identifying the evidence base* by carrying out a systematic review	(i) Reviewed the epidemiological evidence for early life risk factors for obesity. (ii) Improved understanding of the target behaviour. (a) Systematic review of parents' experiences of bottle-feeding to understand how parents decide on quantities and frequency of formula-milk feeds. (b) Systematic review of determinants of early weaning: “Determinants of early weaning and early use of cow's milk” identified determinants of noncompliance with infant feeding recommendations. (iii) Identified existing systematic reviews and checked the controlled trials register for trials of interventions during infancy.

(1.2) *Identifying/developing appropriate theory* by drawing on existing evidence and theory, supplemented if necessary by primary research, for example, interviews/focus groups with “stakeholders”, that is, those targeted by the intervention or involved in its development or delivery	(i) Literature review and team discussions to decide on theory, behaviour change techniques, and intervention strategies. (ii) Qualitative studies with all stakeholders to refine intervention content. These included interviews and focus groups with mothers (recipients of the intervention) and healthcare providers (who would deliver the intervention). In order to optimise the intervention, an iterative process was used with involvement of mothers, behavioural scientists, doctors, midwives, and health visitors.

(1.3) *Modelling process and outcomes* by using a “causal modelling approach” that could include a range of primary and desk based studies to design the intervention, identify suitable measures, and predict long-term outcomes.	(i) Used a causal modelling approach to link “behavioural determinants” to “behavior” and “short-term and long-term outcomes”. (ii) Developed and validated a questionnaire for use in the trial to assess change in key constructs along the causal pathway targeted by the intervention.

(2) *Assessing feasibility and piloting methods *	

(2.1) *Testing procedures* for their acceptability, compliance, and intervention delivery	(i) Tested components independently for feasibility and acceptability and final adaptation of the intervention. (ii) 1 year pilot trial of combined intervention components.

(2.2) *Estimating recruitment and retention* and identifying potential barriers to these, using a mixture of qualitative and quantitative methods	(i) Recruitment through post-natal wards, GPs, Health Visitors, midwives, pharmacies, NHS database, charities, and the media to identify most efficient and effective methods. (ii) Pilot trial over 1 year.

(2.3) *Determining sample size* by anticipating the effect sizes in a pilot study	Pilot trial was too small and no previous trials in this area hence used data from observational studies to estimate sample size.

(3) *Evaluating a complex intervention *	

(3.1) *Assessing effectiveness* by using a randomised controlled trial where possible, choosing the primary and a range of secondary outcomes, and collecting data on predictors or mediators of effect and any possible adverse effects	Set up explanatory RCT (ISRTCN number 2081469). Primary outcome is growth-related and data on a number of secondary outcomes along the causal pathway are also collected. Weight faltering in the babies and reduced quality of life in mothers monitored real time as potential adverse effects reported to independent data monitoring committee.

(3.2) *Understanding change processes* provide insights into why an intervention fails unexpectedly or why a successful intervention works and how it can be optimised. Process evaluation nested within a trial can be used to assess fidelity and quality of intervention delivery, clarify causal mechanisms, and identify contextual factors associated with variations in outcomes. Process evaluations should be conducted to the same high standards and reported just as thoroughly as evaluation of outcomes	(i) Intervention fidelity assessment using prespecified checklists. (ii) Qualitative study nested within the trial-individual interviews with mothers in the intervention and control groups and intervention facilitators to explore how feeding decisions are made, how the intervention might work (or why it may not work) and can be optimised, to identify key ingredients that could be included in future interventions and other contextual factors. (iii) Mediation analyses to understand how the intervention achieved any effects.

(3.3) *Cost-effectiveness analyses* should be included if at all possible, so that the results are useful to decision makers	Cost-consequence analysis planned and data collection on health service utilisation and maternal quality of life in addition to cost of delivering the intervention.

(4) *Implementation and beyond *	

(4.1) *Dissemination* by publication in peer-reviewed literature and also communication with policy makers	Peer reviewed publications, conference presentations, public engagement activities, newsletters, and open access web deposition at the end of the trial.

(4.2) *Surveillance, monitoring, and long-term outcomes *to measure rare or long-term impacts, using routine data sources and record linkage or by recontacting participants	Consent to recontact participants and access routinely collected health and anthropometry data. If intervention is shown to be effective, process and outcome data could inform a future pragmatic trial.

**Table 2:** Behaviour change techniques and intervention strategies used in the baby milk intervention [44].

Technique^a^	Definition^a^	Intervention strategies
(1) Provides information on consequences	Information about the benefits and costs of action or inaction, focusing on what will happen if the person performs the behaviour.	Leaflet explains link between feeding behaviours, rapid weight gain and risk of obesity. This information is reinforced and participant understanding about the information checked during 3 face-to-face and 2 telephone contacts.

(2) Prompts intention formation	Encouraging the person to decide to act or set a general goal.	Leaflet encourages lower guidelines for formula-milk feeding and suggests a general feeding plan. Develop a personalised feeding plan (PFP) in intervention contacts.

(3) Prompts barrier identification	Identifying barriers to performing the behaviour and plan ways of overcoming them.	Identify barriers using cost-benefit analysis, motivation ruler and confidence ruler. Formulation of “if…then…” plans to overcome barriers for example, crying between feeds (“If she cries at night, then I will offer her a dummy”)

(4) Prompts facilitator identification	Identifying facilitators to performing the behaviour and plan ways to use them to overcome barriers.	Cost-benefit analysis, motivation ruler and confidence ruler.

(5) Provides general encouragement	Praising or rewarding the person for effort or performance without this being contingent on specified behaviours or standards of performance.	Praise all attempts at following guidelines. Good communication skills: building rapport, empathy, active listening, nonjudgemental, and client-centred.

(6) Sets graded tasks	Setting easy task and increasing difficulty until target behaviour is performed.	Monthly contact to encourage mothers to set small achievable goals and revise them. Review of personal feeding plan (PFP) to revise goals.

(7) Provides instruction	Telling a person how to perform certain behaviour and/or preparatory behaviours.	Two leaflets and discussion about recommended feeding behaviours during 3 face-to-face and 2 telephone contacts.

(8) Models or demonstrates the behaviour	An expert shows the person how to correctly perform behaviour for example, in class or on video.	Demonstrate the correct method of formula-feed preparation at baseline visit.

(9) Prompts specific goal setting	Involves detailed planning of what the person will do, including a definition of the behaviour specifying frequency, intensity, or duration and specification of at least one context, that is, where, when, how, or with whom.	Personal Feeding plan with goals negotiated with the participant. Make these goals specific by formulating “if…then…” plans

(10) Prompts review of behavioural goals	Review and/or reconsideration of previously set goals or intentions	Review and revise goals set at each intervention contact using the Personal Feeding plan.

(11) Prompts self-monitoring	The person is asked to keep a record of specified behaviour(s) (e.g., in a diary).	Encourage participants to record amount fed in the Personal Feeding plan.

(12) Provides feedback on performance	Providing data about recorded behaviour or evaluating performance in relation to a set standard or others' performance, that is, the person received feedback on their behaviour.	Provide feedback on feeding behaviour, based on Personal Feeding plan. Provide feedback on baby's growth plotted on growth charts.

(13) Teaches to use prompts or cues	Teaching the person to identify environmental cues that can be used to remind them to perform a behavior, including times of day or elements of contexts.	Stickers on formula-milk tins which encourage lower formula-milk consumption.

^a^Labels and definitions of the behaviour change techniques are as specified in Abraham and Michie's Taxonomy of Behaviour Change Techniques [50].

**Table 3:** Intervention and Control contacts and content.

Timeline	Intervention group (IG)	Control group (CG)
First: face-to-face. Within 14 weeks of birth	(i) Healthy growth and nutrition leaflet. (ii) Stickers for formula-milk packets/tins with new guideline daily requirements. (iii) Education about growth charts, rapid weight gain, obesity risk. (iv) Personal feeding plan (PFP). (v) Model feed preparation if necessary.	(i) Standard Department of Health bottle feeding leaflet. (ii) General questions about formula-milk feeding, information sources, and decisions.

Second: telephone. 3-4 months (3–6 weeks later)	(i) Check understanding of key messages. (ii) Review of PFP and goal setting.	General questions about sleep and support with caring for baby.

Third: face-to-face (IG)/telephone (CG) 4-5 months (3–6 weeks later)	(i) Feedback on growth. (ii) Weaning advice. (iii) Review of PFP and goal setting.	General questions about life after the baby's birth.

Fourth: telephone. 5-6 months (3–6 weeks later)	Review of PFP and goal setting.	General questions about formula-milk changes and weaning

Fifth: face-to-face. 6-7 months (3–6 weeks later)	(i) Feedback on growth. (ii) Review of PFP and goal setting.	(i) Standard Department of Health weaning leaflet. (ii) Questions about experience of taking part in the study and research in general.

Identification of barriers and facilitator, problem solving, and “If…then plans” are used in all contacts. All contacts are underpinned by good communication skills. The motivation ruler and confidence ruler are used for assessment and to prompt identification of barriers and facilitators. The “cost-benefit analysis” tool is used as required to improve motivation and confidence.
